# Detecting Gunshots Using Wearable Accelerometers

**DOI:** 10.1371/journal.pone.0106664

**Published:** 2014-09-03

**Authors:** Charles E. Loeffler

**Affiliations:** Department of Criminology, University of Pennsylvania, Philadelphia, Pennsylvania, United States of America; University of Iowa, United States of America

## Abstract

Gun violence continues to be a staggering and seemingly intractable issue in many communities. The prevalence of gun violence among the sub-population of individuals under court-ordered community supervision provides an opportunity for intervention using remote monitoring technology. Existing monitoring systems rely heavily on location-based monitoring methods, which have incomplete geographic coverage and do not provide information on illegal firearm use. This paper presents the first results demonstrating the feasibility of using wearable inertial sensors to recognize wrist movements and other signals corresponding to firearm usage. Data were collected from accelerometers worn on the wrists of subjects shooting a number of different firearms, conducting routine daily activities, and participating in activities and tasks that could be potentially confused with firearm discharges. A training sample was used to construct a combined detector and classifier for individual gunshots, which achieved a classification accuracy of 99.4 percent when tested against a hold-out sample of observations. These results suggest the feasibility of using inexpensive wearable sensors to detect firearm discharges.

## Introduction

Gun violence remains a persistent problem in U.S. communities [1]. Each year, nearly 10,000 individuals are murdered with firearms [2]. The traditional police response to this social problem, increased deployment to suppress gunfire, is difficult to sustain in today's fiscal climate. Therefore, a number of jurisdictions have turned to technological solutions, such as citywide acoustical gunshot location systems, hoping these systems could enhance their ability to detect or deter gun offenders. Unfortunately, these systems have not yet proven to be effective at reducing outdoor gun violence and, by design, are unable to locate indoor gun use [3]. Other jurisdictions have focused their enforcement efforts on monitoring the subset of individuals at highest risk of involvement in fatal shootings based on research showing that the majority of both homicide victims and perpetrators are on probation, parole, or pretrial release [4]–[6]. However, this approach can be labor-intensive. Furthermore, existing monitoring technologies (e.g., RF/GPS bracelets) can lead to information overload for officers [7], [8] in the absence of clear signals that the monitored individuals are at the location of a reported gun crime, which occurs in less than half of all outdoor gun discharges [3]. While the scale of this problem suggests the need for a range of policy responses, the present analysis suggests that an opportunity exists for advanced offender monitoring technology, using low-cost wearable sensors, to enhance public safety by detecting illegal firearm usage by individuals already under the supervision of the criminal justice system.

Prior work on gunshot detection has either focused on shooter localization using acoustic triangulation [9] or localization of muzzle flashes using infrared cameras [10]. In the domestic application, acoustic triangulation has been the most common implementation, with distributed microphone networks constructed to provide location information for gunshot events in covered areas [3].

This approach was disfavored in the current application due to the difficulty of separating handgun-generated muzzle blasts from other impulsive acoustical events, such as jack-hammering,—even at close range—and the challenges of attributing a localized muzzle blast to the wearer of a sensor. Instead, a motion-based detection framework was chosen.

In related work, researchers have achieved success using wearable accelerometers to detect and classify commonplace human behaviors [11]–[15]. In addition, they have recently demonstrated the potential of wearable accelerometers to detect fall events [16], seizures [17], and concussive head trauma [18] from a continuous stream of movement data. To date, however, no published effort has been made to use these sensors to classify firearm use. As a result, our only knowledge of the forces acting upon the wrist during gunshot events comes from studies of firearm movement in controlled settings using load sensors [19], studies of human physiological response to blast pressure and other recoil-related forces [20], and calculations of felt recoil under theoretical conditions [21]. Even when detailed investigations have been made of the firearm-human system, most studies have analyzed shoulder-fired weapons rather than hand-fired weapons and have examined their effects on shooter performance rather than shooter kinematics [20], [22].

Even so, these studies provide several important insights into the physics of firearm use, which aid in the classification of wrist movement during gunshot events. First, gunshot events, from the perspective of the shooter's wrist, occur when the wrist is either at rest or in constant acceleration in the fraction of a second before discharge. This results from the need to aim the gun in the direction of fire. Once the trigger is pulled, there is a sudden change in acceleration. This jerk motion has little in common with other human-initiated activities, which generally involve the gradual acceleration of the wrist prior to peak acceleration (e.g., tennis swings, hand claps, punches, hammering). Instead, this motion has much more in common with other impulse transfer events that involve the hand and arm. These include the hand being struck by a fast moving blunt force object or a high-speed tool. The relative rarity of these impacts will likely provide much of the separation needed to reliably detect gunshots. However, when these confusable events do occur, they are unlikely to overlap substantially with gunshots due to the following three components of gun discharges.

### 1. Blast wave

**Figure 1:**
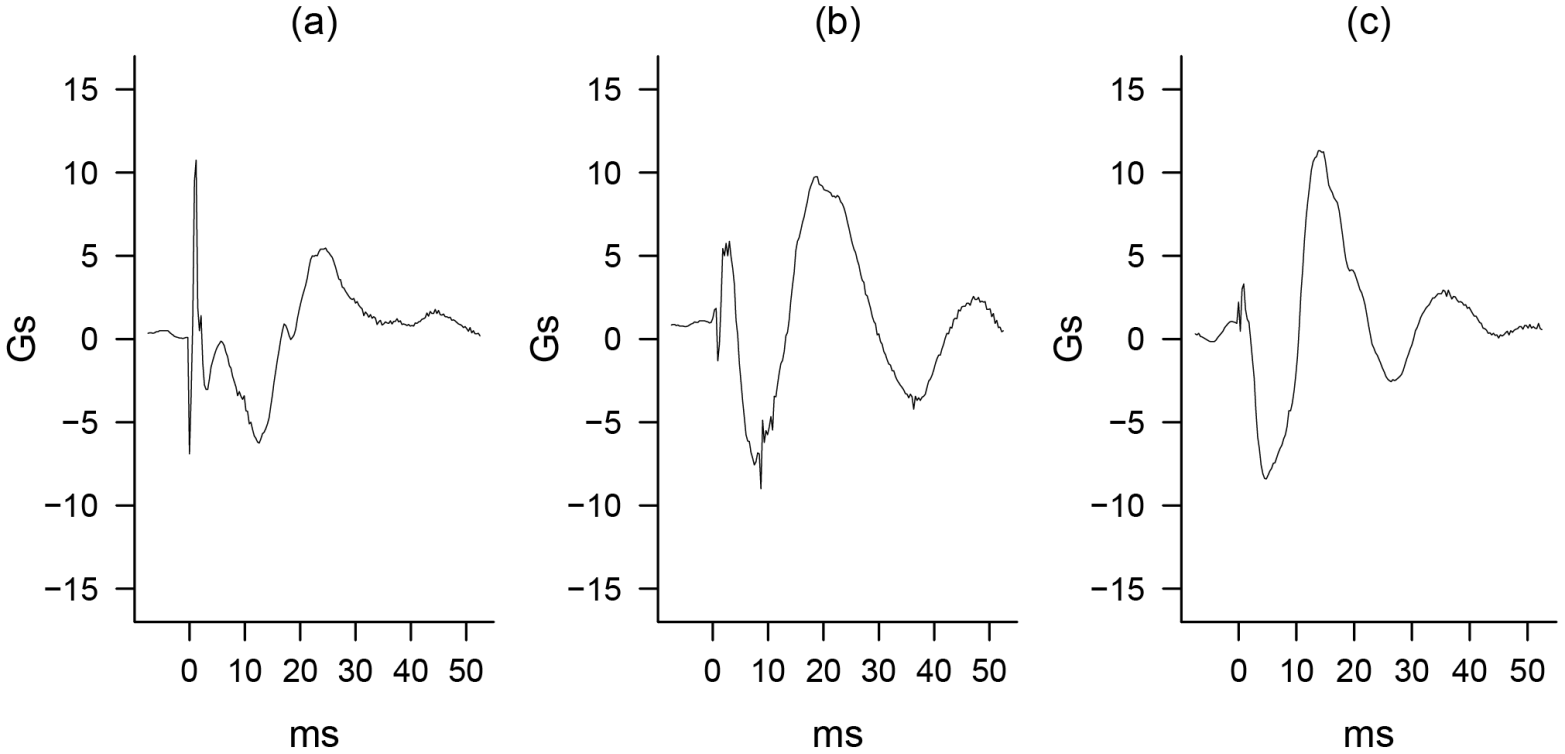
Wrist-Measured Gunshot Example (.38 caliber). Acceleration along the (a) X-axis, (b) Y-axis, and (c) Z-axis.

Only 30% of the chemical energy in a bullet's propellant cartridge is converted to kinetic energy that is transferred to the projectile [23]. This means that the majority of the cartridge propellant is released in the gaseous muzzle blast mixture that accompanies the projectile's exit from the firearm barrel. The resulting muzzle blast, generated by the collision of the rapidly expanding gases with the slower moving air surrounding the barrel of the firearm, forms a spherically propagating blast wave. This blast over-pressure/under-pressure wave will degrade into an acoustical wave within a short distance [24], [25]. However, prior to doing so, it will be detectable as a sudden spike in amplitude when recorded by acoustical microphones and as a sudden spike in acceleration when recorded using microelectromechanical systems (MEMs) accelerometers. An example of such a gunshot-generated blast wave is visible at 0 seconds in Figure 1, with the clearest wave pattern present on the X-axis corresponding to acceleration along the forearm from wrist to elbow.

### 2. Recoil

Recoil of hand-held firearms results from the transfer of energy and momentum from the propellant to the cartridge case to the firearm breach and then finally into the hand and arm. This rearward impulse can be substantial, but it is only applied for the length of time that the projectile remains in the firearm barrel. This is roughly one ms in duration, assuming an average bullet speed of 123.4 m/s and a barrel length of 12.7 centimeters [21]. For the purposes of the present application, the key attribute of recoil is likely to be the timing of this rearward impulse rather than its magnitude, which likely overlaps with other impulsive events. This impulse will not immediately act upon the wrist. Instead, a small delay will precede the transmission of this impulse to the wrist as the gun compresses the soft tissue of the hand. The estimated length of this delay for a shoulder-fired weapon, derived from high speed photography, is approximately 20 to 50 ms [22]. Given the reduced soft tissue on the hand relative to the shoulder, it is likely that this delay will be even shorter for hand-fired weapons. This sequence of events is subject to slight alteration if the weapon being fired contains a recoil absorbing mechanism, such as a semi-automatic pistol. For an example of peak recoil force, see Figure 1 at approximately 14 ms on the X-axis.

### 3. Muzzle lift

During the period when the projectile is moving forward but still within the barrel of the firearm, the rearward pressure of the shell casing on the breach of the firearm is pushing the firearm backwards. Since the center of mass of the firearm is below the plane of the barrel, this force also generates a rotational force around the center of mass. The center of mass is located somewhere along the arm of the shooter, with the exact location being determined by the degree of joint lock and bracing. This rotational force is what generates muzzle lift. In practice, there are only small amounts (fractions of an inch) of upward movement of the weapon-human system and therefore of the wrist [21]. Still, like recoil, this motion occurs over a short period of time and is therefore likely to be observable as a single-positive-peak in the vertical plane occurring at or just after peak recoil. Like rearward recoil, it will occur in too short a time for the human body's neuromuscular system to respond [22]. An example of peak lift can be seen at approximately 18 ms in Figure 1 on the Y-axis.

Additional features of gunshot events and related wrist motions, such as those seen in the Z-axis of Figure 1 (corresponding to acceleration through and perpendicular to the palm of the hand), will also contribute to the classification of gunshot events.

## Materials and Methods

Recruitment consisted of three different subject pools. The first subject pool included ten officers from the University of Pennsylvania Police Department who were asked to participate in a shooting task with six handguns that ranged from a .22 caliber weapon to a .45 caliber weapon. Shot load varied from 36 grains to 230 grains, depending on the weapon caliber. Weapon mass varied from 0.70 to 1.10 kilograms. Weapons included a Rexio Arms .22 caliber revolver, a Smith & Wesson .38 caliber (Model 6) revolver, a Taurus semi-automatic 9 mm pistol (PT99AF), two different Smith & Wesson M&P .40 caliber semi-automatic pistols, and a Colt .45 caliber semi-automatic pistol (MK4 1911). These subjects were instructed to discharge each weapon at a target roughly twenty feet in front of them using both one-handed and two-handed shooting grips. No further instructions on weapon grip were given. While most subjects used standard weapon grips, at least one subject discharged weapons with a wrist rotation of approximately 45 degrees from vertical. In addition, one of the subjects was left-handed, resulting in the inclusion of dominant-hand and non-dominant-hand shooters. The second subject pool consisted of three members of the general population who were asked to engage in their normal routine life activities from morning until evening. Finally, a sample of five construction workers were recruited and asked to engage in their normal construction and demolition tasks, including use of pneumatic nailguns, pneumatic jack-hammers, .22 caliber powder-actuated fastener guns, and other construction tools. The University of Pennsylvania's Institutional Review Board (Protocol 818910) approved this study, and each participating subject completed a written informed consent form.

Each subject was fitted on their right wrist with a wearable tri-axis accelerometer [AX3 Watch, Axivity Ltd.], which is capable of recording acceleration up to 16 Gs at a rate of 3.2 khz (or 3 times per ms) for extended periods (≥12 hours). Police subjects wore the sensors for the duration of their shooting task (∼20 minutes). Two construction workers engaged in demolition of concrete wore the sensors for two hours each, and the three remaining construction workers engaged in framing work wore the sensors for 6 hours. The three other control subjects wore the sensors for 6 to 8 hours at a time. Once the data collection was completed, the subjects were split into training/validation and test samples. Sensor data from five police officers, three construction workers and two non-construction control subjects formed the training/validation sample. Data from the remaining five police officers, two construction workers and the final non-construction control subject formed the test sample. All sensor data is available online (http://dx.doi.org/10.7910/DVN/25918).

**Figure 2:**
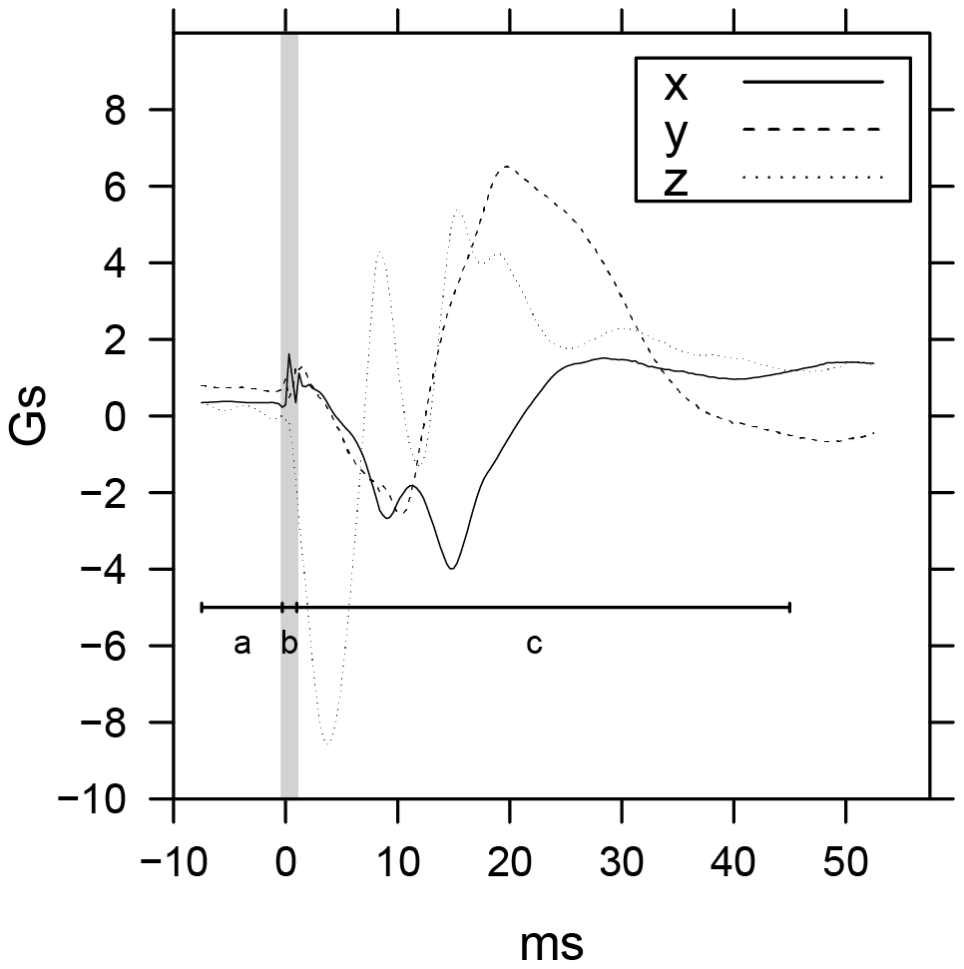
Sample Averaged Gunshots with Feature Windows. Tri-axis accelerometer readings for 359 aligned and averaged gunshots. Windows for the calculation of statistical features cover the period from −7.5 ms pre-spike to 45 ms post-spike. Window (a) covers the pre-spike period from −7.5 ms to −0.3 ms, window (b) covers the spike period from −0.3 ms to 1 ms, and window (c) covers 1 ms to 45 ms.

The logged sensor data from both samples, consisting of over 68 hours of recordings, were pre-processed using a sliding-window spike detector algorithm to identify all candidate ballistic spikes. Each candidate spike was defined as any 1.5 G magnitude increase on the X-axis over a 600 µs period preceded by a magnitude increase of less than 1.5 G on the X-axis over the previous 600 µs period. An example of a candidate spike can be seen in Figure 1(a) at 0 ms. In practice, this simple detector procedure identified the leading edge of each possible spike and greatly reduced the number of candidates that needed to be classified. For each candidate ballistic spike, of which there were 2787 in all, feature windows covering the period from 7.5 ms pre-spike to 45 ms post-spike were then constructed with a pre-spike window (a) covering −7.5 ms to −0.3 ms, a spike window (b) covering −0.3 ms to 1 ms, and a post-spike window (c) covering 1 ms to 45 ms (Figure 2). Feature statistics were then calculated for each candidate spike.

Given the nature of the gunshot event, all feature statistics were calculated in the time-domain. Statistical features were designed to capture different aspects of the tri-axis gunshot patterns observed in Figure 2. These included the pre-spike sum of the differences between the overall measured acceleration vector and the acceleration due to gravity, which captured the amount of pre-blast spike activity. For the blast spike, spike magnitude and change in magnitude were calculated. The post-spike patterns observed in Figure 2 were captured by a number of different features including the peak recoil acceleration value, the distance between the spike feature and the peak recoil acceleration, peak lift value and its distance from the spike feature. Pre-spike and spike statistical features were calculated using raw sensor data, while post-spike window features were computed using smoothed sensor data filtered by locally-weighted regression methods to eliminate high frequency noise [26]. This filtering method estimates a non-parametric regression surface similar to moving average calculations.

**Figure 3:**
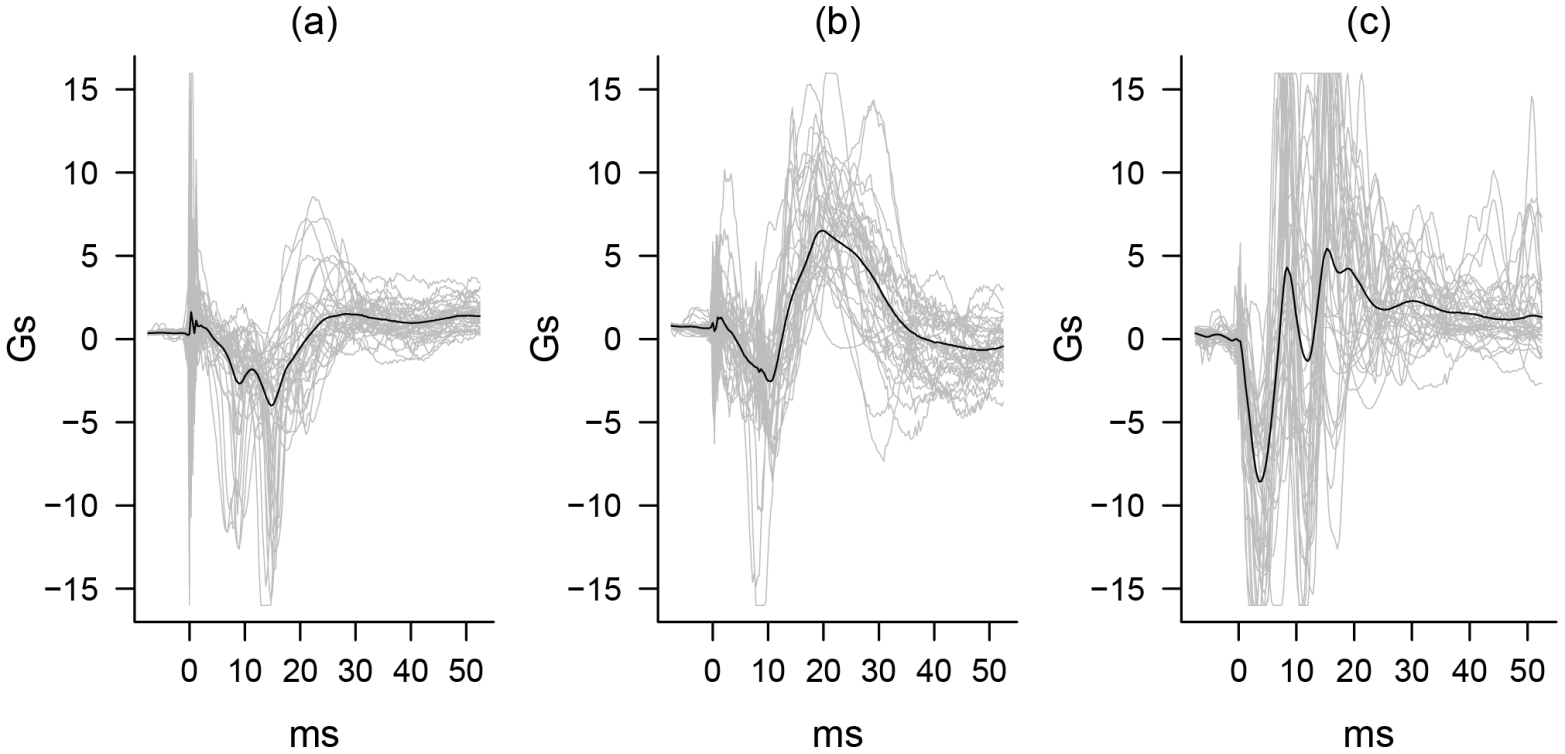
Gunshot Features with Averages. Individual and averaged gunshot acceleration readings along the (a) X-axis, (b) Y-axis, and (c) Z-axis. Individual gunshot acceleration readings (in grey) are a 10 percent sample of the 359 gunshot acceleration readings (in black) in the sample average.

Due to the variation in the recorded signal (Figure 3) resulting from sensor filtering, shooter bracing, and variability in firearm mechanisms (e.g., revolver versus semi-automatic), a probabilistic classification method was selected over a deterministic decision-rule approach. All statistical features for each candidate spike were entered into a logistic regression model predicting whether a spike had been labeled as a gunshot or not. Feature selection was accomplished using penalized regression as implemented in the glmnet package in R [27]. This method fits a L1-regularized logistic regression model to the full set of standardized features. This shrinkage variable selection and weighting method was selected because of its ability to handle the highly-correlated features derived from the accelerometer data. Features and corresponding feature weights were selected at the value of regularization parameter (λ) that provided the minimum mean cross-validated classification error in the training/validation sample.

## Results

The resulting model excluded five statistical features and, contrary to expectation, selected feature weights for the remaining features that balanced feature value and location. This procedure achieved a cross-validated classification accuracy of 99.7±0.1 percent in the training/validation sample. Since no gunshots were excluded by the detector, this figure also describes the overall accuracy of the detector-classifier system in the training sample. Inspection of the standardized feature weights of the final model suggested the relative importance of feature values over feature locations. However, the use of a different non-parametric modeling strategy might easily produce a different balance between these two elements of the gunshot signal. In order to verify that the gunshot classifier was not over-fitted to the training sample, the test sample, comprised of the gunshots from the five excluded police officers as well as the spikes from the remaining control subjects, was classified using the trained gunshot classifier. The results of this out-of-sample classification are reported in Table 1. Of 358 gunshots in the test data, 354 were correctly identified by the classifier, three were misclassified, and one was excluded from classification by the candidate detector. In addition, of the 693 confusable spikes, only three were classified as gunshots. This produced an overall sensitivity of 0.989 and a specificity of 0.996 for the detector-classifier system.

**Table 1:** Test Sample Confusion Table.

	Classified Gunshot	Not Classified Gunshot
**Actual Gunshot**	354	3
**Not Actual Gunshot**	3	690

Further analysis revealed that the three false negative misclassifications were similar to their neighboring gunshots except for having larger amounts of pre-spike activity and some cross-axis feature splitting, possibly resulting from sensor frame rotation. Examination of the three false positive misclassifications found evidence of early y-axis peak lift values, excessive pre-spike activity on the z-axis, and prolonged x-axis spikes. All three false negative misclassifications differed visibly from gunshot events.

## Discussion

Consistent with recent advances in human activity recognition [11], [12], [16], the present study investigated the possibility that firearm use could be reliably distinguished from routine human activities and from known confusable activities using MEMs inertial sensor technology. The results suggest that accelerometer-based classification of firearm use is feasible and could form the basis of a wearable and affordable gunshot detection sensor system.

The apparent success of this recognition methodology likely results from several of the peculiar features of firearm usage. Unlike other impulsive events generated by the human body, firearm usage begins with an essentially stationary body, which is necessitated by the aiming task. Metal-on-metal collisions generated by the hand, such as hammering a nail, by contrast, are generally preceded by considerable pre-event accelerations. And common impulsive events that happen to the human body, such as a collision or other physical impact, are not accompanied by a preceding blast wave. Similarly, the human-weapon system, having a center of mass below the barrel of the weapon, generates a muzzle lift feature that aids in the classification task. This is most strikingly seen in the successful discrimination of .22 caliber firearm gunshots from .22 caliber powder-driven fasteners, both of which use the same explosive charge. Taken together, these three features contribute to the utility of wrist-measured acceleration as a method for detecting firearm utilization. In addition, given that single gunshots make up only a fraction of all non-self-inflicted illegal firearm discharges, it is likely that a gun use detection system based on the underlying gunshot detector-classifier reported in this paper could have higher overall accuracy.

Before developing such a wearable gun use detection system, which could be implemented as a logging monitor or an event-triggered wireless remote monitor, several limitations of the current classifier system should be addressed. First, implementation of a higher sampling frequency sensor would provide improved capture of the blast wave feature. The current 3.2 khz sensor provided a filtered signal that missed portions of the characteristic ballistic N-wave for some gunshot events. Reduced filtering would enable the use of a more discriminating spike detector while also contributing additional information to the classifier. Second, additional data, including data from additional handguns but especially shoulder-fired weapons, would improve the generalizability of the classifier. This latter class of weapons was omitted from the present study due to the overwhelming use of handguns in gun-related violent crimes. Likewise, testing this classifier on data from non-expert shooters would provide a further assessment of its generalizability. Finally, the possibility of firearm use in the opposite and non-monitored hand would either need to be addressed by use of dual wrist monitors or the development of a second classifier for detecting opposite-wrist observable features (e.g., blast waves).

The potential use of sensors to monitor firearm use among community-supervised offenders also raises important public policy and civil liberties concerns. These include the selection criteria by which judges or other releasing authorities would place individuals on this form of supervision; whether it would be used on the existing community-supervised offender population or on an otherwise incarcerated offender population; the necessary level of sensor system accuracy before sanctions, such as revocation of release, could be imposed; and the procedures that would be taken to minimize the collection of non-firearm-related wearer information. While none of these issues are peculiar to a wearable gunshot detection system, these longstanding concerns regarding offender monitoring systems should be revisited as monitoring technology evolves.

In conclusion, this study suggests that low-cost and low-energy motion sensors can be used to identify firearm discharges. This development offers criminal justice practitioners a potential alternative that overcomes the low signal-to-noise ratio that has characterized many location-based behavioral monitoring tools [7], [8] and community-wide acoustical gunshot monitoring systems [3]. This development would be more in keeping with the experience of remote monitoring technology for detection of substance abuse [28] and the promise that if reliable and low-noise signals of other illegal conduct can be found, such conduct could be reduced through enhanced detection or deterrence [29], [30].
